# Multi-omics characterization of type 2 diabetes mellitus-induced gastroenteropathy in the db/db mouse model

**DOI:** 10.3389/fcell.2024.1417255

**Published:** 2024-08-15

**Authors:** Yuxin Zhang, Yanjiao Zhang, Ruiyang Yin, Xinyi Fang, Runyu Miao, Huifang Guan, Yiqi Yao, Jiaxing Tian

**Affiliations:** ^1^ Institute of Metabolic Diseases, Guang’anmen Hospital, China Academy of Chinese Medical Sciences, Beijing, China; ^2^ Graduate College, Beijing University of Chinese Medicine, Beijing, China; ^3^ Graduate College, Changchun University of Chinese Medicine, Changchun, China

**Keywords:** diabetic gastroenteropathy, mouse, metabolomics, transcriptomics, proteomics

## Abstract

**Objective:**

Gastrointestinal dysfunction are often associated with type 2 diabetes mellitus (T2DM), a complicated metabolic illness. Contributing factors have been proposed, including genetic predisposition, gene environmental, and lifestyle interactions, but the pathophysiology remains unknown.

**Methods:**

We aim to explore the possible causes behind gastrointestinal dysfunction caused by type 2 diabetes in this study. A comprehensive analysis of the gastric sinus metabolome, transcriptome, and proteome in db/db mice with gastrointestinal dysfunction was conducted.

**Results:**

The model group of mice had considerably lower small intestine propulsion and gastric emptying rates, higher blood glucose levels, and were significantly obese compared to the control group. We identified 297 genes, 350 proteins, and 1,001 metabolites exhibiting significant differences between db/db and control mice (*p* < 0.05). Moreover, multi-omics analysis revealed that the genes, proteins, and metabolites in the T2DM-induced gastroenteropathy mice group were involved in arachidonic acid metabolism, glycerophospholipid metabolism and vitamin digestion and absorption. Specifically, Cbr3, Etnppl, and Apob were the major mRNAs associated with T2DM-induced gastrointestinal dysfunction, while Cyp2b10, Cyp2b19, Pgs1, Gpat3, Apoa4, and Tcn2 were the major proteins associated with T2DM-induced gastrointestinal injury, and 16(R)-HET, 5-HETE, LysoPC (22:0), and Pantothenic acid were the major metabolites associated with T2DM-induced gastrointestinal disorders.

**Conclusion:**

The mechanism of action of diabetic gastroenteropathy may be related to vitamin digestion and absorption, glycerophospholipid metabolism, and arachidonic acid metabolism.

## 1 Introduction

Diabetic gastroenteropathy is one of the most prevalent consequences of inadequate blood glucose management. The primary symptoms include weight loss, nausea, vomiting, stomach distension, early satiety, postprandial fullness, and epigastric pain (Meling et al., 2022). The number of adults with diabetes in China has exceeded 140 million, of which more than 50% have delayed gastric emptying (GE) and various gastrointestinal symptoms (Ye et al., 2021; Ogurtsova et al., 2022). Genetic vulnerability to type 2 diabetes has been associated with an elevated risk of a number of gastrointestinal disorders, according to a mendelian randomization study (Chen et al., 2023). The pathophysiology of diabetic gastroenteropathy is still poorly understood, and it will therefore be critically important to investigate the risk factors and preventative strategies of gastrointestinal disorders linked to diabetes.

Research on diabetic gastrointestinal disease now has greater possibilities thanks to the development of omics approaches (transcriptomics, proteomics, metabolomics) and ongoing enhancement of analytical techniques (Song et al., 2022). Synergistic interactions and complementing effects between different types of molecules are not captured by single omics. To investigate the probable mechanism of type 2 diabetes mellitus (T2DM)-induced gastrointestinal disorders, the current work investigated the gastric metabolomes, transcriptomes, and proteomes of diabetic gastrointestinal lesion mice using leptin-deficient db/db mice.

## 2 Materials and methods

### 2.1 Experimental animals

The 6-week-old healthy BKS-db/db mice and wild type (WT) mice were provided by Beijing Pharmacokang Biotechnology Co., LTD (n = 6/group), license number: SYXK (Beijing) 2023–0,032, and were raised in Laboratory Animal Center of Beijing University of Chinese Medicine, Ethics number: BUCM-2023030906–1,109. The experimental room was kept at (22 ± 2)°C in terms of temperature, 60% ± 10% in terms of humidity, regular ventilation, and 12 h of alternating lighting.

### 2.2 Fasting blood glucose and body weight

Throughout the study period, body weight and absolute fasting blood glucose levels were measured using a standard methodology at the conclusion of every other week (Surme et al., 2023).

### 2.3 Measurement of GE rate and intestinal transit

Following an overnight fast, the mice were given 0.1 mL/10 g of 1.5% carboxymethylcellulose (Shanghai yuanye Bio-Technology Co., Ltd., China) containing 0.05% phenol red (Shanghai yuanye Bio-Technology Co., Ltd., China) orally. The mice were sacrificed by cervical dislocation after a further twenty minutes, and their stomachs were meticulously dissected. The gastric contents were added to 100 mL of 0.1 N NaOH and allowed to settle at room temperature for 1 h. A centrifuge was used for 30 min at 3,000 rpm after 5 mL of the solution’s supernatant was added to 0.5 mL of 20% trichloroacetic acid. A microplate reader (Multiskan GO, Thermo Fisher Scientific, United States of America) was used to measure the absorbance of the supernatant at a wavelength of 560 nm after it was combined with 4 mL of 0.05 M NaOH. The GE rate was calculated using the following Equation: Gastric emptying (%) = (Phenol red absorbance–residual phenol red absorbance)/phenol red absorbance × 100%.

The gastric and small intestine were separated to determine the amount of intestinal transit. After being removed in their entirety, the small intestines naturally straightened and were spread out on white filter paper. The distance of phenol red from the pylorus and the total length of the small intestine were determined. Intestinal transit (%) = Phenol red advances the distance/the total length of the small intestine × 100%. Graphpad Prism 9.0 was used to perform two-way ANOVA and *t*-test analyses of the data.

### 2.4 Transcriptomics analysis of gastric tissue

Total RNA was extracted from 100 mg of gastric tissue samples from each mouse (n = 6/group)using the TRIzol (thermofisher, 15,596,018) reagent according to the manufacturer’s instructions and was isolated and purified. The total RNA’s quantity and purity were subsequently assessed using a NanoDrop ND-1000 (NanoDrop, Wilmington, DE, United States of America), and the RNA’s integrity was verified using a Bioanalyzer 2,100 (Agilent, CA, United States of America). The Illumina NovaSeq 6,000 system (Illumina, United States of America) was used to sequence RNA in sequencing mode PE150. After receiving the sequencing raw data, it was processed using APTBIO’s proprietary perl scripts to remove adaptor sequences and filter out low-quality or N-ratio reads more than 5%. Clean data was then aligned to the mice reference genome using Hisat2 (version 2.2.1), and reads corresponding to each gene were counted using FeatureCounts.

Differential expression analysis was performed using the DESeq2 R package (version 1.22.2) (Liu et al., 2021). The threshold was set at *p*-value <0.05 and log2 fold change (log2FC) > 1 in absolute value. Heatmaps were used to illustrate the differences in expression between samples after clean data were uploaded to the NCBI database in order to identify significantly differentially expressed genes (DEGs). The screened differential genes were subjected to Kyoto encyclopedia (KEGG) pathway analysis (http://www.genome.jp/kegg/), and histograms were used to show the significant enrichment of the DEGs and regulatory pathways.

### 2.5 Proteomics analysis of gastric tissue

100 mg of gastric tissue per mouse was lysed in SDT buffer and its content was determined by BCA method (n = 6/group). Proteins were labeled following enzymolysis in accordance with the TMT labeling kit’s instructions, and data were gathered using nanoLC-MS/MS. For each sample, 200 ng of total peptides were separated and analyzed with a nano-UPLC (nanoElute2) coupled to a timsTOF Pro2 instrument (Bruker) with a nano-electrospray ion source. Separation was performed using a reversed-phase column (PePSep C18, 1.9 𝜇, 75 𝜇 × 25 cm, Bruker, Germany). Mobile phases were H2O with 0.1% FA (phase A) and ACN with 0.1% FA (phase B). Separation of sample was executed with a 60 min gradient at 300 nL/min flow rate. Gradient B: 2% for 0 min, 2%–22% for 45 min, 22%–37% for 5 min, 37%–80% for 5 min, 80% for 5 min. The mass spectrometer adopts DDA PaSEF mode for DDA data acquisition, and the scanning range is from 100 to 1700 m/z for MS1. During PASEF MS/MS scanning, the impact energy increases linearly with ion mobility, from 20 eV (1/K0 = 0.6 V s/cm2) to 59 eV (1/K0 = 1.6 V s/cm2).

Vendor’s raw MS files were processed using SpectroMine software (4.2.230,428.52329) and the built-in Pulsar search engine. MS spectra lists were searched against their species-level UniProt FASTA databases (uniprot-Mus musculus-reviewed-10090–2022–11. fasta), Carbamidomethyl [C] as a fixed modification, Oxidation (M) and Acetyl (Protein N-term) as variable modifications. A maximum of two missed cleavage(s) was allowed. For both PSM and peptide levels, the false discovery rate (FDR) was set to 0.01. A fragment mass deviation of 20 ppm and an initial precursor mass deviation of up to 20 ppm were used for peptide identification. Standard settings applied to all other parameters. Once the anti-database has been created, import the DIA data, apply the FDR filter using the mProphet algorithm, then utilize the MSstats R language package to get the quantitative results related to protein. Following the standardization of the quantitative results differentially expressed proteins (DEPs) (fold change >1.2 or fold change <0.83 and *p*-value <0.05) were filtered out for further bioinformatics investigations using statistical analysis. The Student’s t-test was used to verify the differences in proteins between the groups were significant.

### 2.6 Metabolomics analysis of gastric tissue

#### 2.6.1 Metabolites Extraction

Each mouse gastric tissue (n = 6/group) weighing 25 mg was subjected to 500 μL of extraction solution containing internal standard (methanol: acetonitrile: water = 2:2:1 V/V/V), vortex mixed, and then subjected to a 4-min grinding treatment at 35 Hz followed by a 5-min sonication (in an ice water bath). Following three repetitions of the aforementioned steps, −40°C was allowed to stand for 1 hour. After that, the sample was centrifuged for 15 min at 4°C at 12,000 rpm. For analysis, the resultant supernatant was moved to a fresh glass vial. An equal amount of the supernatants from each sample was combined to create the quality control (QC) sample.

#### 2.6.2 LC-MS/MS Analysis

After then, LC-MS/MS analyses were performed using a UHPLC system (Vanquish, Thermo Fisher Scientific) with a Phenomenex Kinetex C18 (2.1 mm × 50 mm, 2.6 μm) coupled to Orbitrap Exploris 120 mass spectrometer (Orbitrap MS, Thermo).

Data preprocessing and annotation: ProteoWizard was used to convert the raw data to mzXML format, which was then processed by BiotreeDB (V2.1) self-built secondary mass spectrometry database for peak detection, extraction, alignment, and integration that was written in R (version 3.3.5) and based on XCMS. Then, the algorithm score’s cutoff value is set to 0.3 after the material annotation matches in the database that was created by the user for secondary mass spectra.

#### 2.6.3 Data integration analysis

The resulting dataset, which included the sample name, peak number, and normalized peak area, was loaded into the SIMCA16.0.2 multivariate analysis software package (Sartorius Stedim Data Analytics AB, Umea, Sweden). For group separation and identifying significantly changed metabolites, supervised orthogonal projections to latent structures-discriminate analysis (OPLS-DA) was applied, followed by a 7-fold cross validation. Permutation tests (200 times) were conducted to examine the robustness and predictive power of the OPLS-DA model. Following that, the *R* ^2^ and Q^2^ intercept values were determined. The Q^2^ intercept value represents the model’s robustness, risk of overfitting, and dependability, with smaller values indicating better performance. OPLS-DA analysis revealed the variable importance in projection (VIP) score of the first principal component. Metabolites with VIP>1 and p < 0.05 were classified as significantly altered. To control the false discovery rate (FDR), *p*-values were revised using the Benjamini–Hochberg (BH) multiple testing adjustment. To ensure system stability and data reliability, three quality control (QC) samples were produced by pooling 10 μL of each stomach sample and analyzed using the same approach. Commercial databases such as KEGG and MetaboAnalyst (http://www.metaboanalyst.ca/) were used to conduct pathway enrichment analysis.

### 2.7 Integrated multi-omics analysis

Based on the screening results of DEGs, DEPs and DEMs, the results of metabolome and transcriptome, metabolome and proteome, and transcriptome and proteome analyses were mapped on KEGG plots, respectively, to further analyze their correlations.

### 2.8 Quantitative real-time PCR

Total RNA was extracted from the gastric tissue using the TRIzol reagent (invitrogen) following the manufacturer’s instructions. The reverse transcription of RNA into cDNA was performed using a HiFiScript cDNA Synthesis kit (CW2596M, CWBIO, Jiangsu, China). An UltraSYBR Mixture (CW2602, CWBIO) was used for qRT-PCR (real-time quantitative PCR). The qRT-PCR results were normalized using beta-actin (ACTB). The relative gene expression levels were analyzed using the 2^−ΔΔCT^ method (Du et al., 2022). Primer sequences were provided in Table 1.

**TABLE 1:** PCR primers for quantitative real-time PCR.

Primer	Forward	Reverse
Cbr3	CTG​GTG​TGG​TCT​GAT​TCT​TTC​C	GCA​GGC​ACA​TTA​ACT​GGT​TGA​A
Etnppl	AGA​CAG​CAA​CTT​TTC​TAT​GCC​TG	CCA​CAG​ACA​AGA​AGT​TCC​CTG​AA
Apob	TAA​AGA​CCA​TCC​TGA​GCC​AGA​C	TCA​TCT​TGA​GTT​CAG​GCT​GCT​T
ACTB	CCT​AGC​ACC​ATG​AAG​ATC​AAG​AT	ACT​CAT​CGT​ACT​CCT​GCT​TGC​T

### 2.9 Western blotting

The total protein was extracted from the gastric tissue using RIPA reagent, and the results were obtained using BCA assay kits. 30 mg of protein samples were electrophoretically separated using a constant voltage of 80 V for the concentration glue and 120 V for the separation glue before being transferred into a PVDF membrane. Incubated for 2 h at room temperature with 5% skim milk powder, then overnight at 4°C with the following primary antibody: Cyp2b10, Cyp2b19, Pgs1, Gpat3, Tcn2, Apoa4 and Tubulin. The secondary antibody was then added and incubated for 2 h. A sufficient amount of ECL chemiluminescent solution was applied for development. Meanwhile, Tubulin was adopted to be the loading control.

## 3 Results

### 3.1 Db/db mice display increased body weights, elevated fasting blood glucose, and gastrointestinal dysfunction

**FIGURE 1:**
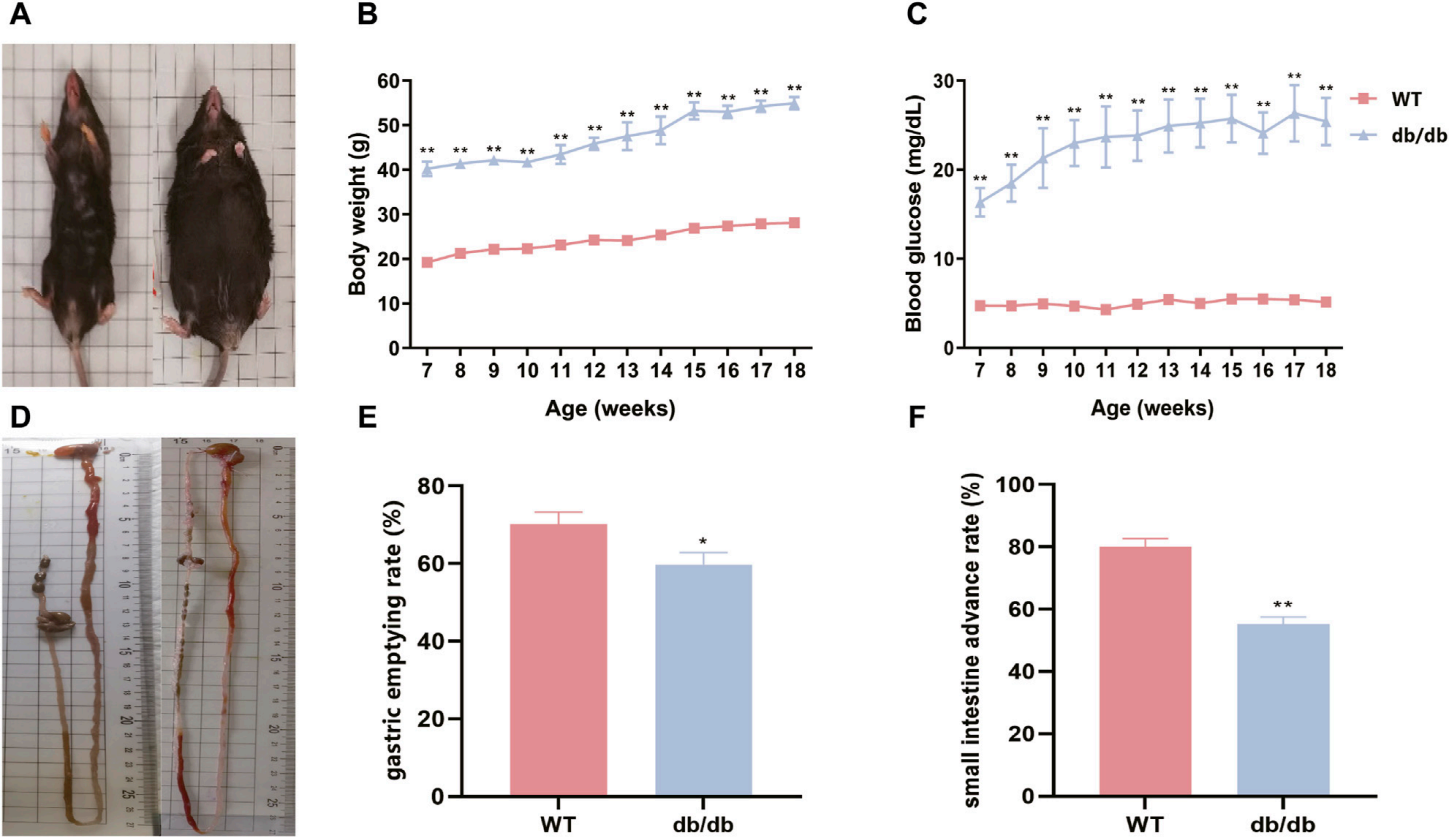
db/db mice display increased body weights, elevated fasting blood glucose, and gastrointestinal dysfunction. **(A)** Gross morphology of mice between two groups (WT mice is on the left, and db/db mice is on the right). **(B)** Body weight curves of two groups. **(C)** Fasting blood glucose curves of two groups. **(D)** Gross morphology of gastric and small intestine between two groups (WT mice is on the left, and db/db mice is on the right). **(E)** Gastric emptying rate of two groups. **(F)** Small intestine advance of two groups. The standard error of the mean is shown by the error bars, while the mean values are represented by the columns (n = 6), *p < 0.05; **p < 0.01.

The db/db mice were noticeably fatter than the WT mice and showed little desire to get active (Figure 1A). The body weight and fasting blood glucose levels of db/db mice were considerably greater than those of WT mice (Figures 1B,C). Furthermore, the db/db mice exhibited a significant decrease in both gastric emptying rate and small intestine advance rate as compared to the WT mice (Figures 1D–F).

### 3.2 Transcriptomic analysis of gastric tissue

**FIGURE 2:**
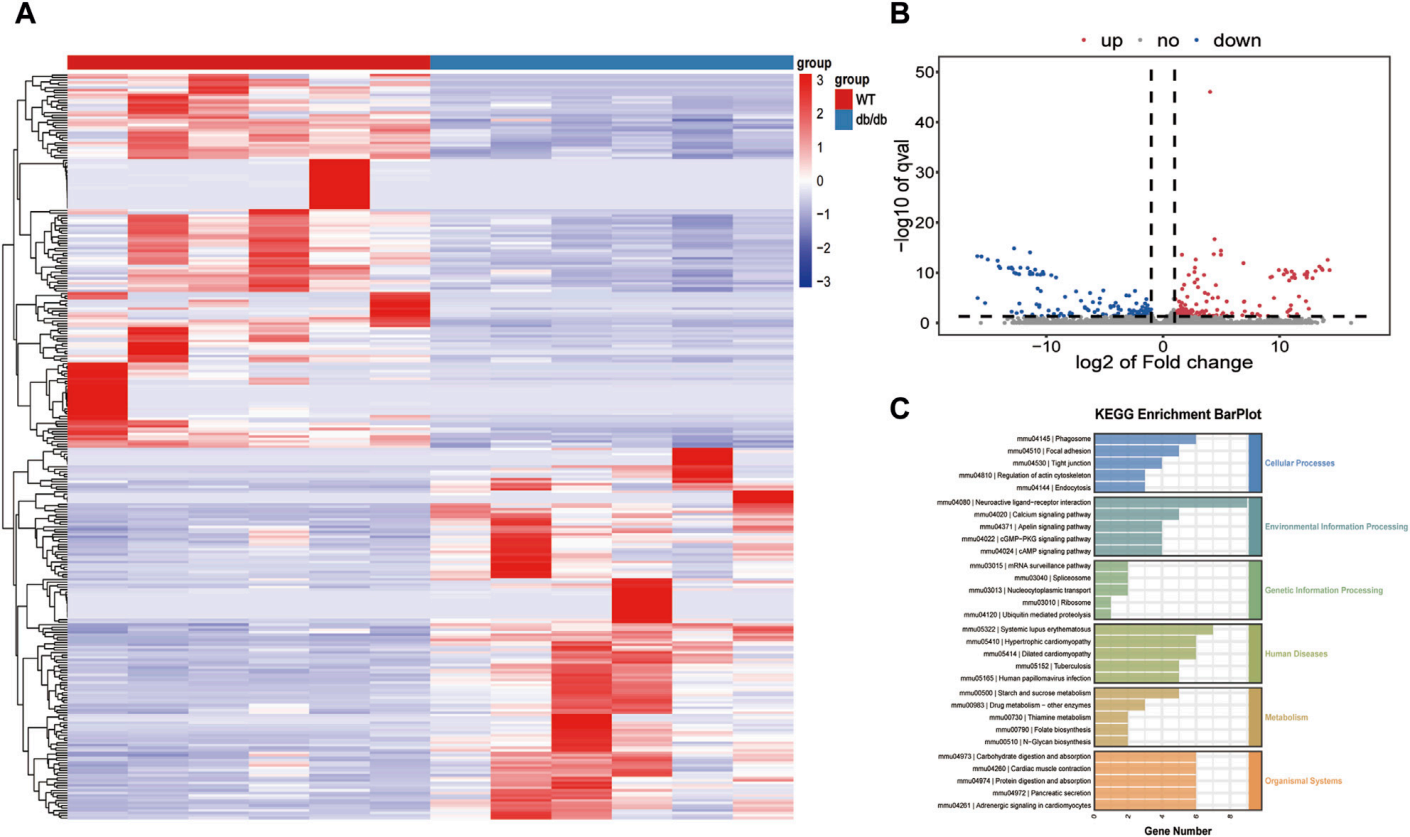
Transcriptomics analysis **(A)** Heatmap of the DEGs. **(B)** Volcano plot of the DEGs. **(C)** KEGG analysis for DEGs.

148 genes were upregulated and 149 genes were downregulated in the db/db group of mice used for transcriptomic analysis, which aimed to investigate the transcriptional basis of the diabetic gastroenteropathy formation process. Additionally, the aforementioned differential genes were shown using heat maps (Figures 2A,B). These 297 DEGs were implicated in several metabolic pathways, such as the cellular processes, environmental information processing, genetic information processing, human diseases, metabolism, and organismal systems. Specifically, these metabolic pathways include neuroactive ligand-receptor interaction, carbohydrate digestion and absorption, and protein digestion and absorption (Figure 2C). Quality control results for the transcriptomic data are shown in Supplementary Table S1, Supplementary Figure S1.

### 3.3 Proteomic analysis of gastric tissue

**FIGURE 3:**
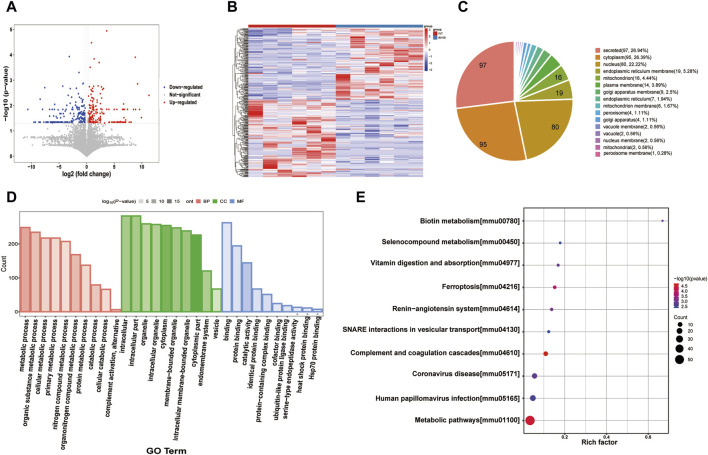
Proteomics analysis **(A)** Volcano plot of the DEPs. **(B)** Heatmap of the DEPs. **(C)** Subcellular distribution for DEPs. **(D)** GO analysis for DEPs. **(E)** KEGG pathway annotation for DEPs.

mRNA expression levels do not correspond to protein levels because of post-transcriptional and post-translational regulation. Proteins are the primary effector molecules that ultimately take part in cell biological processes since they are the primary executors of cell functions. Quality control results for the proteomics data are shown in Supplementary Figure S2. The total ion chromatogram (TIC)/base peak chromatogram (BPC) based on LC-MS has good overlap, indicating high repeatability and stability of the chromatography. A total of 6,212 proteins and 52,360 peptides were identified based on Label-free proteomics technology. A total of 350 DEPs between the control and model groups were identified through protein identification and difference analysis by quantitative proteomics, of which 168 were upregulated and 182 were downregulated (Figures 3A,B).

**FIGURE 4:**
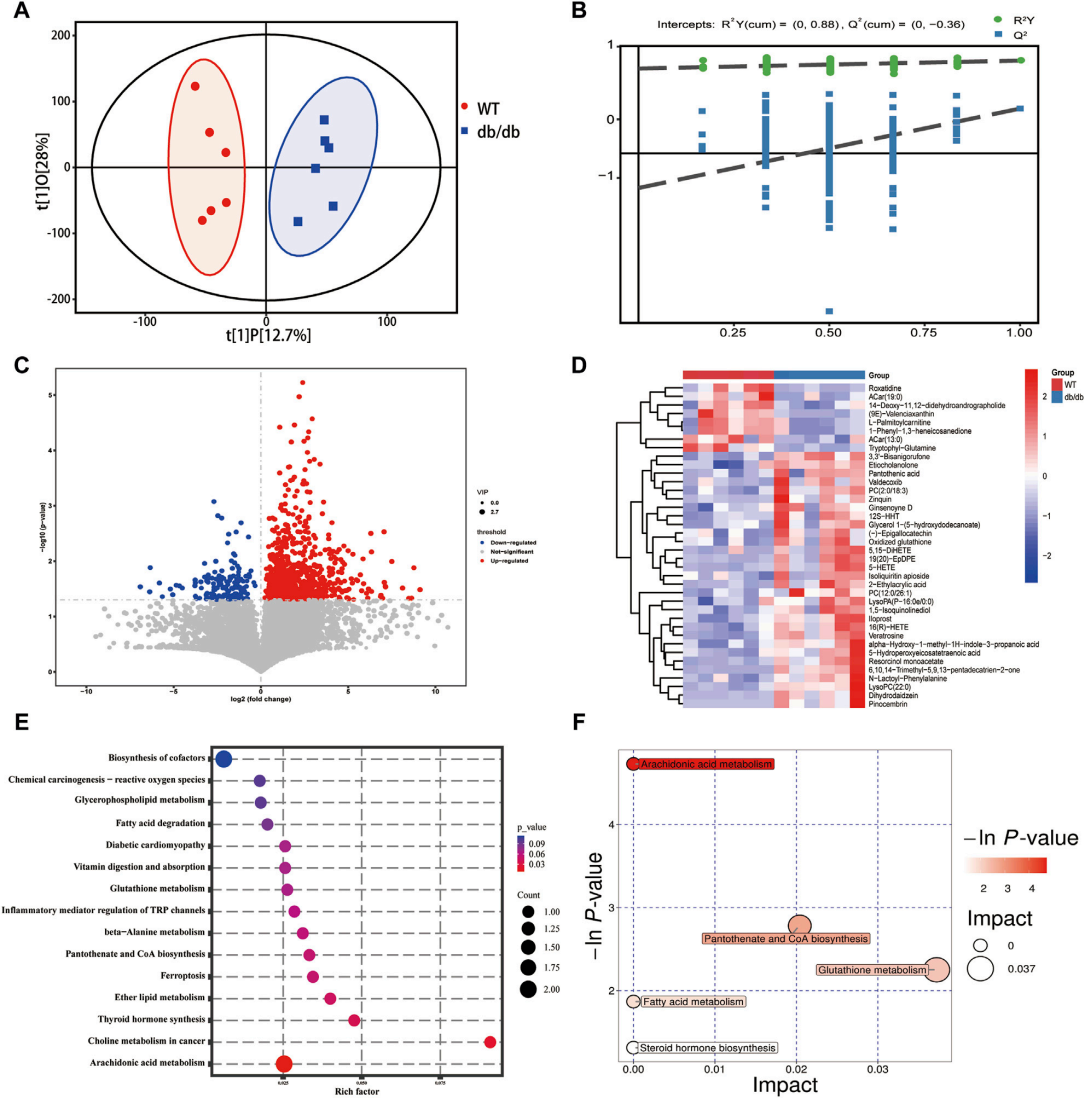
Metabolomics analysis **(A)** Score scatter plot of OPLS-DA model for group WT vs db.db. **(B)** Permutation plot test of OPLS-DA model for group WT vs db.db. **(C)** Volcano plot of the DEMs. **(D)** Heatmap of the DEMs. **(E)** KEGG analysis for DEMs. **(F)** Pathway analysis for group WT vs db/db.

Subcellular localization refers to the precise location within a cell where a protein or gene expression product is present. Pie charts were used to represent the results of subcellular localization investigations of differentially expressed proteins in order to precisely comprehend protein structure, characteristics, and relationships (Figure 4C). DEPs function mainly in regions such as secreted, cytoplasm, nucleus, endoplasmic reticulum membrane, and mitochondrion.

GO Enrichment Biological Process (BP) includes metabolic process, organic substance metabolic process, cellular metabolic process, and primary metabolic process. Intracellular, intracellular part and organelle are all considered components of the cellular component (CC). Molecular Function (MF) includes binding, protein binding, and catalytic activity. (Figure 3D). For these 25 DEPs, functional enrichment analysis was carried out. KEGG pathways are mainly enriched in metabolic pathways, vitamin digestion and absorption, ferroptosis, SNARE interactions in vesicular transport and other signaling pathways (Figure 3E).

### 3.4 Metabolomics analysis of gastric tissue

The OPLS-DA approach with supervision was used to maximize the separation between groups and validate the separation of diverse mouse samples in the WT group and db/db treatment group. The OPLS-DA (Figure 4A) clearly shows the separation of the samples from each group, and the two mice groups’ gastric metabolic profiles were all found to be within the 95% confidence interval. Furthermore, it was discovered that the *R* ^2^ value for the OPLS-DA model was greater than the Q^2^ value, and the Q^2^ regression line had a negative intercept (*R* ^2^ = [0, 0.88], Q^2^ = [0, −0.36]), indicating that the model is meaningful (Figure 4B).

Three within-run quality control (QC) samples in total were used to assess the metabolomic analytical system’s repeatability. The QC samples’ spectral peak overlaps, as depicted in Supplementary Figure S3, were within very small variations, and the QC samples’ correlation is nearly 1, demonstrating the UPLC-Q-TOF/MS system’s strong stability and great data quality.

The expression of 1,001 endogenous metabolites was significantly altered in the db/db group compared with the WT group, of which 851 metabolites were upregulated and 150 metabolites were downregulated (Figure 4B). To visualize the relative amounts of possible biomarkers in each sample, heat maps were generated against the relative intensities of the metabolites (Figure 4C), where redder colors corresponded to higher levels of the differential metabolite and bluer colors to lower levels. KEGG pathway enrichment analysis of all differential metabolites showed that the KEGG pathways enriched mainly included arachidonic acid metabolism, pantothenate and CoA biosynthesis, glutathione metabolism, fatty acid metabolism, steroid hormone biosynthesis and vitamin digestion and absorption (Figures 4D,E).

### 3.5 Integrated multi-omics analysis

**FIGURE 5:**
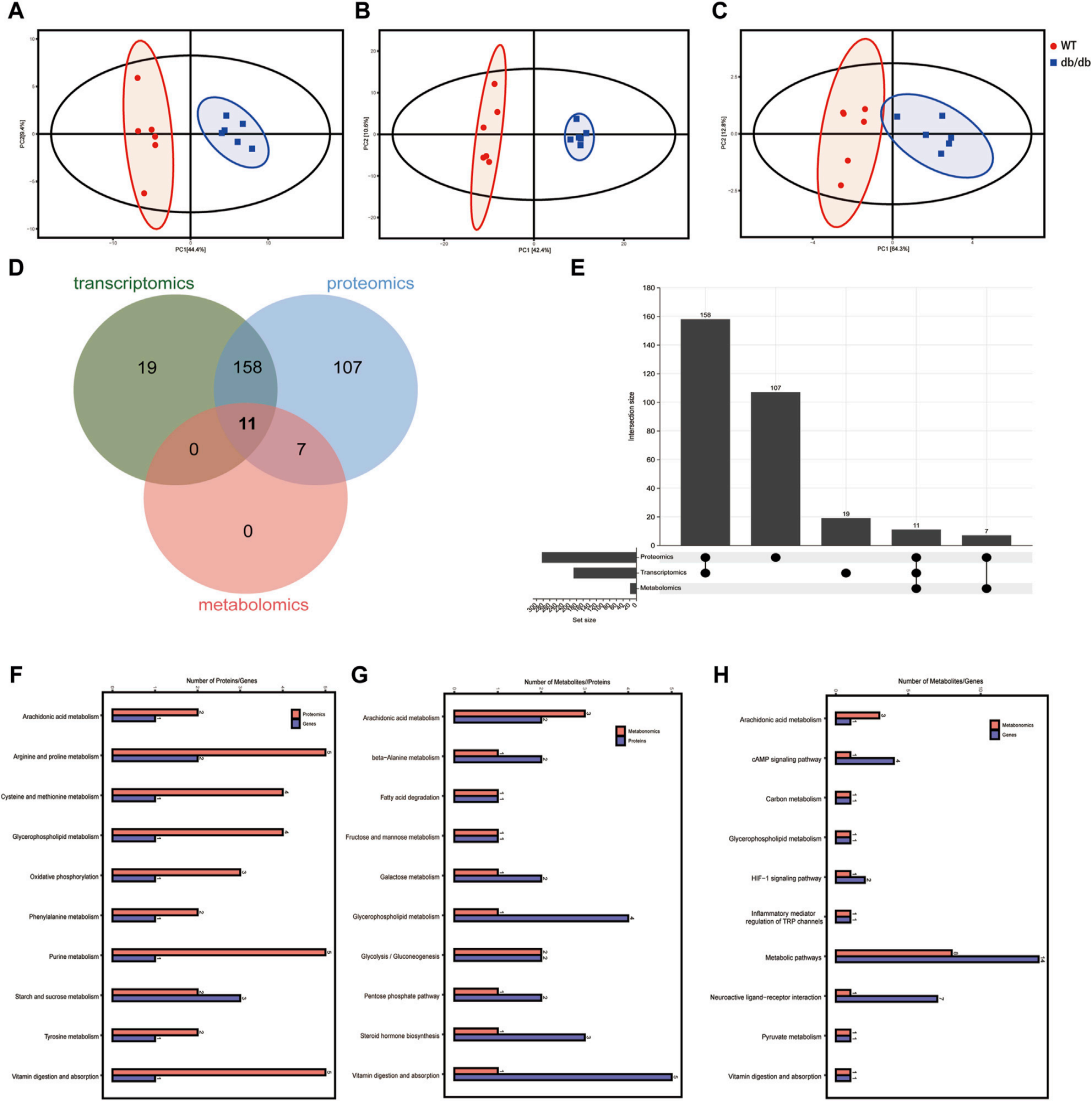
Integrated multi-omics analysis. **(A)** PCA score plot of the transcriptomics in positive mode. **(B)** PCA score plot of the proteomics in positive mode. **(C)** PCA score plot of the metabolomics in positive mode. **(D)** Venn diagram of the KEGG pathway shows the participation of transcriptomics, proteomics and metabolomics from group WT compared to group db/db. **(E)** Upset plot of the KEGG pathway shows the participation of transcriptomics, proteomics and metabolomics from group WT compared to group db/db. **(F)** The common pathways in both transcriptomics and proteomics. **(G)** The common pathways in both proteomics and metabolomics. **(H)** The common pathways in both transcriptomics and metabolomics.

Based on the transcriptomic, metabolomic and proteomic data, the degree of aggregation of samples in the two groups and the overall distribution trend of samples between the groups were observed by PCA method. The results showed that the two groups could be clearly separated (Figures 5A–C), the transcriptomics horizontal coordinate PC1 accounted for 44.4% of the sample difference, and the vertical coordinate PC2 accounted for 9.4% of the sample difference; the proteomics horizontal coordinate PC1 accounted for 42.4% of the sample variation and PC2 in vertical coordinate accounted for 10.6% of the sample variation; and the metabolomics horizontal coordinate PC1 indicated the first principal component, which accounted for 64.3% of the sample difference, and the vertical coordinate PC2 indicated the second principal component accounted for 12.8% of the sample difference.

According to the previous article (Lu et al., 2023; Zou et al., 2024), Venn diagram and upsetplot (Figures 5D,E) were plotted to establish the KEGG pathways and the number of KEGG pathways that differed between the groups in terms of metabolite, gene and protein co-involvement, and the results showed that there were a total of 11 transcriptomics-proteomics-metabolism shared pathways, 169 transcriptomics-proteomics shared pathways, and 18 proteomics-metabolism shared pathways. KEGG pathway enrichment cluster analysis diagrams can be found in Figures 5F–H and Table 2. Multi-omics analysis revealed that key genes, proteins and metabolites from T2DM-induced gastroenteropathy mice group were involved in arachidonic acid metabolism, glycerophospholipid metabolism and vitamin digestion and absorption.

**TABLE 2:** Information on key genes, proteins, metabolites, and pathways.

KEGG pathway	Key gene	Key protein	Key metabolites
Arachidonic acid metabolism	Cbr3	Cyp2e1, Cyp2b10, Cyp2b19	16(R)-HET, 5-HETE
Glycerophospholipid metabolism	Etnppl	Pld3, Cept1, Pisd, Pgs1, Gpat3	LysoPC(22:0)
Vitamin digestion and absorption	Apob	Apoa4, Apoa1, Pnlip, Tcn2	Pantothenic acid

**FIGURE 6:**
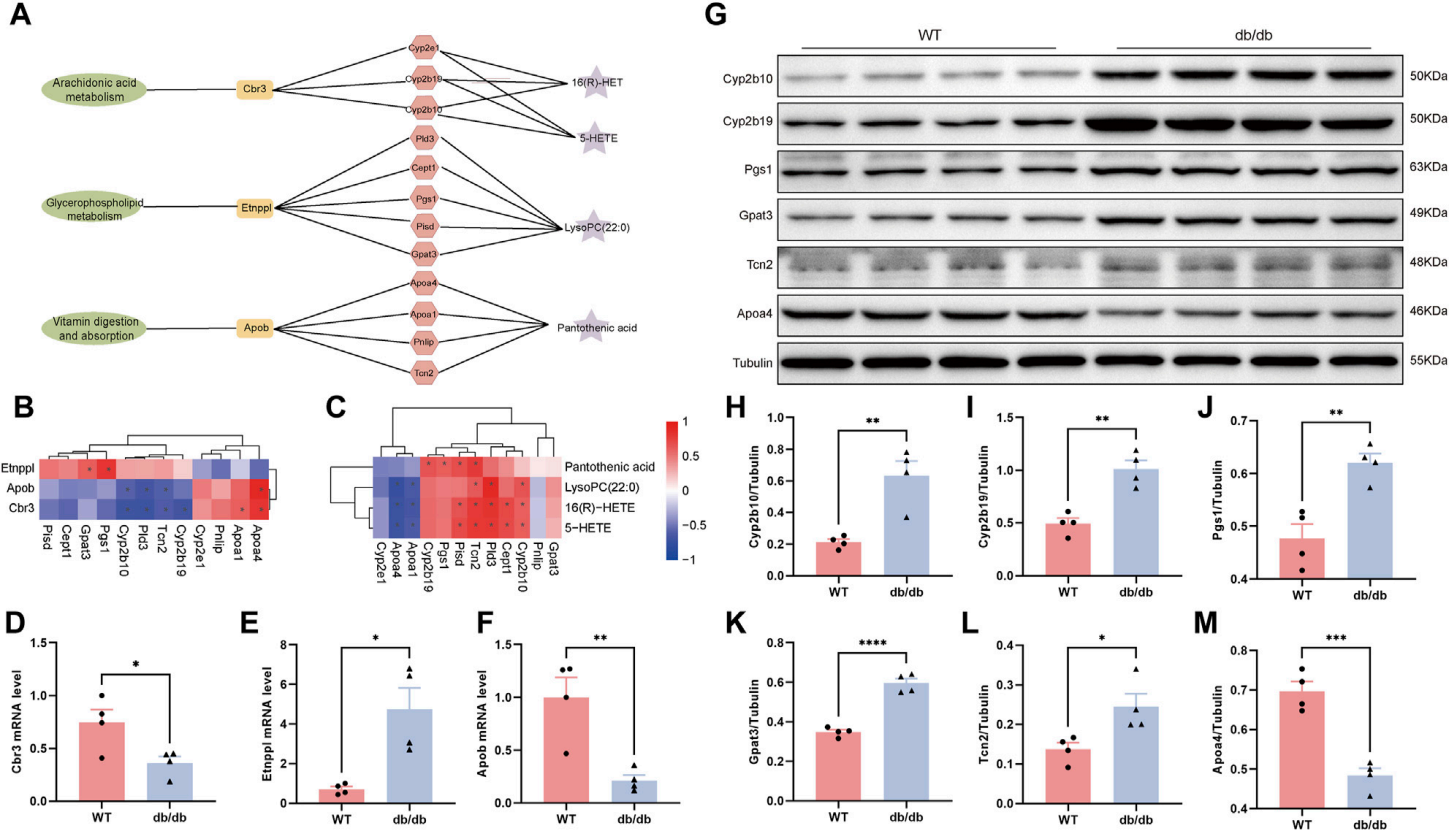
Integrated analysis of multi-omics of transcriptomics, proteomics and metabolomics in db/db mice. **(A)** Pathway-genes-proteins-metabolites network node diagram. **(B)** Heatmap showing correlation of key gene and key protein. **(C)** Heatmap showing correlation of key protein and key metabolites. *p < 0.05 for correlation coefficient significance test, red represented positive correlation and blue represented negative correlation. The mRNA levels of Cbr3 **(D)**, Etnppl **(E)** and Apob **(F)**. **(G)** Relative protein expression levels of Cyp2b10 **(H)**, Cyp2b19 **(I)**, Pgs1 **(J)**, Gpat3 **(K)**, Tcn2 **(L)** and Apoa4 **(M)** by Western blotting. Results were presented as mean ± SEM (n = 4). *p < 0.05, **p < 0.01, ***p < 0.001, ****p < 0.0001 versus respective control.

Evaluation of correlation between key genes, proteins and metabolites using Spearman’s correlation coefficient and mapping of metabolite-gene-protein network nodes. In the Arachidonic acid metabolism pathway, Cbr3 is negatively correlated with Cyp2b10 and Cyp2b19, and Cyp2b10 is positively correlated with 16(R)-HET and 5-HETE. In the Glycerophospholipid metabolism pathway, ethanolamine phosphate phospholyase (Etnppl) was positively correlated with Pgs1 and Gpat3, and Pld3 was positively correlated with LysoPC(22:0). Apob was negatively connected with Tcn2 and favorably correlated with Apoa4 in the pathway of Vitamin digestion and absorption. Furthermore, there was a positive correlation between Tcn2 and Pantothenic acid (Figures 6A–C; Supplementary Tables S2, S3).

Based on the combined analysis of multi-omics, we carried out Real-Time PCR and Western blot to verify the expression changes of differential mRNA and proteins. Compared with the WT control group, the expressions of Cbr3 and Apob mRNA in db/db group decreased, and the expressions of Etnppl mRNA increased (Figures 6D–F). Consistent with the findings of the comprehensive analysis, the Cyp2b10, Cyp2b19, Pgs1, Gpat3, and Tcn2 protein expressions were significantly greater and Apoa4 protein expression was significantly reduced in the db/db group as compared to the WT group (Figures 6G–M).

## 4 Discussion

Diabetic gastroenteropathy is one of the most common diabetes complications. Gastric motility abnormalities and delayed gastric emptying brought on by metabolic diseases affecting the gastrointestinal nerves and muscles are the primary pathogenic characteristics (Petri et al., 2021). Diabetes modifies the motor activity of specific stomach regions, resulting in abnormal gastric emptying patterns (Goyal, 2021). Hormone levels, anomalies in the neuro-endocrine system, and the lack or destruction of Cajal interstitial cells are all directly linked to diseases of the T2DM-Induced gastrointestinal motility disorders (Zhang et al., 2024). The pathogenesis is still unclear, so this study intends to explore the possible molecular mechanisms of diabetic gastroenteropathy by combined multi-omics techniques.

BKS-db/db leptin-deficient mice with spontaneous hyperglycemia, insulin resistance, and obesity symptoms are ideal models for studying type II diabetes and its complications (Han et al., 2023). Variations in blood glucose levels influence glucose-stimulated or glucose-inhibited neurons in gastric inhibitory vagal circuits or gastric excitatory vagal circuits, which leads to changed levels of gastric emptying (Grover et al., 2019). In this study, it was observed that mice in the 18-week model group had significantly higher blood glucose and significantly lower gastric emptying rate and small intestine advance rate compared with the control group, suggesting that the mouse model of diabetic gastroenteropathy was successfully constructed.

In this study, analysis of the co-enrichment pathways of DEMs, DEPs, and DEGs revealed three co-enrichment pathways of arachidonic acid metabolism, glycerophospholipid metabolism, and vitamin digestion and absorption. During biological reactions, fatty acids are digested and produced as energy substrates and play a role in the methodical control of physical activity (Kimura et al., 2020). Arachidonic acid (AA) belongs to the Omega 6 family of long-chain polyunsaturated fatty acids and is an essential polyunsaturated fatty acid released from cell membrane phospholipids and metabolized through the cyclooxygenase, cytochrome P450 (CYP450) and lipoxygenase. CYP450-derived arachidonic acid metabolites is a critical key to understanding the pathogenesis of diabetes, dysglycolipid metabolism, and delayed gastric emptying (Bashashati et al., 2021; Isse et al., 2022). AA metabolites play a role in controlling apoptosis, cell proliferation, metabolism and esterification of cholesterol, increasing vascular elasticity and decreasing blood viscosity after binding to the corresponding receptors and triggering downstream signaling pathways in different tissues (Wang et al., 2019; Zhou et al., 2021). Previous studies have also shown that the hub metabolites of gastrointestinal injury are mainly closely related to arachidonic acid metabolism and vitamin metabolism (Morse et al., 2019; Zhou et al., 2019). Carbonyl reductase 3 (Cbr3) is essential for the metabolism of endogenous substances, catalyzing the reduction of carbonyls to hydroxyls, and may also protect against oxidative stress by reducing reactive carbonyl species, maintaining metabolic homeostasis, and participating in cellular antioxidant defense systems (Blanc et al., 2021). In this study, we showed that in the arachidonic acid metabolic pathway, Cbr3 gene expression was downregulated in the gastric sinus tissue of the diabetic gastroenteropathy model group, indicating the presence of oxidative stress and abnormalities in endogenous chemical metabolism, as well as potential damage to critical proteins and metabolites. Cyp2b10 and Cyp2b19 are members of the CYP450 enzyme family (Gethings et al., 2021), in T2DM-induced gastrointestinal diseases, Cbr3 negatively regulates the expression of Cyp2b10 and Cyp2b19 proteins, resulting in elevated protein expression. Moreover, proteomic and metabolomic association analyses showed that Cyp2b10 has a positive correlation with 16(R)-HET and 5-HETE in T2DM-induced gastroenteropathy.

Glycerophospholipid (GPL) synthesis and breakdown represent one of the most closely regulated metabolisms during the 24-h cycle in terms of total lipid content, enzyme expression, and activity in the nervous system and individual cells (Guido et al., 2022). Numerous diseases can result from GPL metabolic problems, which can also cause insulin resistance, obesity, dyslipidemia, endoplasmic reticulum stress, and other metabolic abnormalities (Chen et al., 2022). This study revealed a close relationship between abnormal glycerophospholipid metabolism and gastrointestinal lesions associated with diabetes. Additionally, the expression levels of the Etnppl gene, Pld3, Cept1, Pisd, Pgs1, Gpat3 proteins, and LysoPC(22:0) compounds were found to be upregulated in the gastric tissues of mice in the model group. Etnppl regulates phosphatidylethanolamine (PE) homeostasis by degrading phosphoethanolamine. PE is an essential component of cell membranes, keeping them fluid and functional. Etnppl activity may influence PE production and cell membrane structure, affecting neuronal cell function (White et al., 2021). In the present study, Etnppl gene was found to be positively correlated with Pgs1 and Gpat3 protein in T2DM -induced gastroenteropathy, with elevated levels of Pgs1 and Gpat3 protein expression. The creation of cellular energy and other metabolic processes depend on mitochondrial biogenesis and function, which are indirectly supported by PGS1’s activity in the endoplasmic reticulum (Li et al., 2024). GPAT3 is a crucial component of lipid metabolism and is located in the endoplasmic reticulum, and studies have revealed that Gpat3 deficiency regulates oxidative stress (Fan et al., 2024). We speculate that gastrointestinal injury in diabetic mice may be mediated by upregulating the expression of the Etnppl gene and Pgs1 and Gpat3 proteins, thereby regulating mitochondrial dysfunction in cells.

Abnormal digestion and absorption of vitamins are closely associated with the development of diabetic gastroenteropathy, and previous studies have shown that vitamin deficiency is a risk factor for gastroparesis in patients with type 2 diabetes, and Tcn2 is a transporter protein for vitamin B12 (Ahmed et al., 2023). Through mitigation of oxidative stress and maintenance of the cholinergic contractile responses in the fundus and pylorus, vitamin C demonstrated a significant therapeutic effect on gastric emptying dysfunction in diabetic rats (Da Silva et al., 2017). After the vitamin enters the red blood cells, it is transformed into pyridoxal phosphate, which aids in the metabolism of proteins, fats, and carbohydrates and is crucial for the synthesis of 5-hydroxytryptophan, which helps patients with diabetic gastroparesis who are experiencing vomiting to stop (Li, 2019). In the gastric tissues of T2DM gastroenteropathy, the current study showed downregulation of Apob gene expression, upregulation of Tcn2 protein expression, downregulation of Apoa4, Apoa1, and Rnlip protein expression, and upregulation of key metabolite expression of Pantothenic acid in the pathway of vitamin digestion and absorption. Apoa4 mediates the regulation of food intake and satiety through the central system of the rat hypothalamus (Qu et al., 2019). Additionally, Spearman’s correlation and WB, PCR experiments were used to further validate the relationship between genes and proteins, and it can be found that Apob gene is positively correlated with and Apoa4 protein, and negatively correlated with Tcn2 protein.

There is some limitation in this study. Although genes, proteins, metabolites, and pathways closely linked to the pathogenesis of diabetic gastroenteropathy have been identified, additional research is required to confirm this finding. As a result, we will confirm the expression levels of important proteins and metabolites in the subsequent studies in the next plan.

## 5 Conclusion

In summary, certain impacts of chronic hyperglycemia on gastric metabolism and protein synthesis in mice have been observed, based on the findings of transcriptome, proteomics, and metabolomics study. The mechanism of action of diabetic gastrointestinal lesions may be closely related to the digestion and absorption of vitamins, glycerophospholipid metabolism, and arachidonic acid metabolism pathway by applying the BKS-db/db spontaneous diabetic mouse model.

## Data Availability

The datasets presented in this study can be found in online repositories. The names of the repository/repositories and accession number(s) can be found in the article/Supplementary Material.
