# Supplementary material for: Multi-omics characterization of type 2 diabetes mellitus-induced gastroenteropathy in the db/db mouse model
Source: Front Cell Dev Biol. 2024 Aug 15;12:1417255. doi: 10.3389/fcell.2024.1417255 (PMC11357919; doi:10.3389/fcell.2024.1417255)
Supplement: Supplementary file 1 [file Table1.DOCX]

**Table S1** Reliability evaluation of gastric transcriptomics in WT and db/db mice.

| **Sample** | **Valid data read** | **Valid ratio** | **Q20%** | **Q30%** | **GC content%** |
| --- | --- | --- | --- | --- | --- |
| **WT1** | 53205412 | 96.69 | 99.45 | 96.70 | 52.00 |
| **WT2** | 39038764 | 92.18 | 99.05 | 94.92 | 44.00 |
| **WT3** | 36783642 | 90.20 | 98.97 | 94.78 | 44.00 |
| **WT4** | 36298194 | 89.04 | 98.94 | 94.63 | 44.00 |
| **WT5** | 36443200 | 88.42 | 98.97 | 94.80 | 44.00 |
| **WT6** | 51882750 | 96.02 | 99.33 | 95.75 | 50.00 |
| **db/db1** | 38248376 | 94.27 | 99.01 | 96.11 | 43.00 |
| **db/db2** | 35221698 | 89.12 | 99.02 | 94.88 | 43.00 |
| **db/db3** | 50435930 | 96.92 | 99.51 | 97.10 | 52.50 |
| **db/db4** | 52950152 | 96.59 | 98.64 | 95.39 | 50.00 |
| **db/db5** | 47341460 | 91.56 | 99.69 | 97.44 | 53.00 |
| **db/db6** | 35150964 | 92.18 | 98.97 | 96.01 | 43.50 |

**Table S2** Spearman correlation analysis between key genes and key proteins.

| **Key gene** | **Key protein** | **Corr** | **Corr.p** |
| --- | --- | --- | --- |
| Apob | Apoa4 | 0.8811 | 0.0002 |
| Apob | Apoa1 | 0.5804 | 0.0521 |
| Apob | Cyp2e1 | 0.4865 | 0.1088 |
| Apob | Pnlip | 0.3310 | 0.2933 |
| Cbr3 | Apoa4 | 0.8042 | 0.0027 |
| Cbr3 | Apoa1 | 0.6084 | 0.0400 |
| Cbr3 | Cyp2e1 | 0.4039 | 0.1929 |
| Cbr3 | Pnlip | 0.5211 | 0.0823 |
| Etnppl | Pld3 | 0.3287 | 0.2974 |
| Etnppl | Cyp2b19 | 0.1744 | 0.5877 |
| Etnppl | Tcn2 | 0.3536 | 0.2594 |
| Etnppl | Cyp2b10 | 0.3453 | 0.2716 |
| Etnppl | Cept1 | 0.4578 | 0.1345 |
| Etnppl | Pgs1 | 0.8071 | 0.0015 |
| Etnppl | Pisd | 0.5188 | 0.0839 |
| Etnppl | Gpat3 | 0.6150 | 0.0333 |

**Table S3** Spearman correlation analysis between key proteins and key metabolites.

| **Key protein** | **Key metabolites** | **Corr** | **Corr.p** |
| --- | --- | --- | --- |
| Pld3 | Pantothenic acid | 0.5455 | 0.0027 |
| Pld3 | LysoPC(22:0) | 0.8881 | 0.0378 |
| Pld3 | 16(R)-HETE | 0.8182 | 0.0839 |
| Pld3 | 5-HETE | 0.7972 | 0.2511 |
| Cyp2b19 | Pantothenic acid | 0.6609 | 0.0047 |
| Cyp2b19 | LysoPC(22:0) | 0.5324 | 0.0428 |
| Cyp2b19 | 16(R)-HETE | 0.5599 | 0.0925 |
| Cyp2b19 | 5-HETE | 0.5691 | 0.2720 |
| Tcn2 | Pantothenic acid | 0.7780 | 0.0047 |
| Tcn2 | LysoPC(22:0) | 0.6324 | 0.0428 |
| Tcn2 | 16(R)-HETE | 0.7822 | 0.0921 |
| Tcn2 | 5-HETE | 0.7988 | 0.2717 |
| Apoa4 | Pantothenic acid | -0.4406 | 0.0027 |
| Apoa4 | LysoPC(22:0) | -0.7273 | 0.0378 |
| Apoa4 | 16(R)-HETE | -0.7063 | 0.0839 |
| Apoa4 | 5-HETE | -0.6923 | 0.2516 |
| Cyp2b10 | Pantothenic acid | 0.2080 | 0.0047 |
| Cyp2b10 | LysoPC(22:0) | 0.6698 | 0.0428 |
| Cyp2b10 | 16(R)-HETE | 0.6074 | 0.0921 |
| Cyp2b10 | 5-HETE | 0.6157 | 0.2717 |
| Apoa1 | Pantothenic acid | -0.3706 | 0.0029 |
| Apoa1 | LysoPC(22:0) | -0.6224 | 0.0384 |
| Apoa1 | 16(R)-HETE | -0.6503 | 0.0839 |
| Apoa1 | 5-HETE | -0.6154 | 0.2516 |
| Cyp2e1 | Pantothenic acid | -0.4589 | 0.0047 |
| Cyp2e1 | LysoPC(22:0) | -0.5048 | 0.0428 |
| Cyp2e1 | 16(R)-HETE | -0.3855 | 0.0918 |
| Cyp2e1 | 5-HETE | -0.3855 | 0.2665 |
| Pnlip | Pantothenic acid | 0.0704 | 0.0040 |
| Pnlip | LysoPC(22:0) | -0.2465 | 0.0406 |
| Pnlip | 16(R)-HETE | -0.1972 | 0.0887 |
| Pnlip | 5-HETE | -0.2042 | 0.2660 |
| Cept1 | Pantothenic acid | 0.4155 | 0.0040 |
| Cept1 | LysoPC(22:0) | 0.5423 | 0.0406 |
| Cept1 | 16(R)-HETE | 0.7183 | 0.0873 |
| Cept1 | 5-HETE | 0.7042 | 0.2616 |
| Pgs1 | Pantothenic acid | 0.6157 | 0.0047 |
| Pgs1 | LysoPC(22:0) | 0.4868 | 0.0428 |
| Pgs1 | 16(R)-HETE | 0.5242 | 0.0925 |
| Pgs1 | 5-HETE | 0.5159 | 0.2720 |
| Pisd | Pantothenic acid | 0.6830 | 0.0046 |
| Pisd | LysoPC(22:0) | 0.5001 | 0.0428 |
| Pisd | 16(R)-HETE | 0.6681 | 0.0890 |
| Pisd | 5-HETE | 0.7054 | 0.2665 |
| Gpat3 | Pantothenic acid | 0.1010 | 0.0047 |
| Gpat3 | LysoPC(22:0) | 0.4039 | 0.0428 |
| Gpat3 | 16(R)-HETE | 0.3396 | 0.0936 |
| Gpat3 | 5-HETE | 0.2754 | 0.2720 |


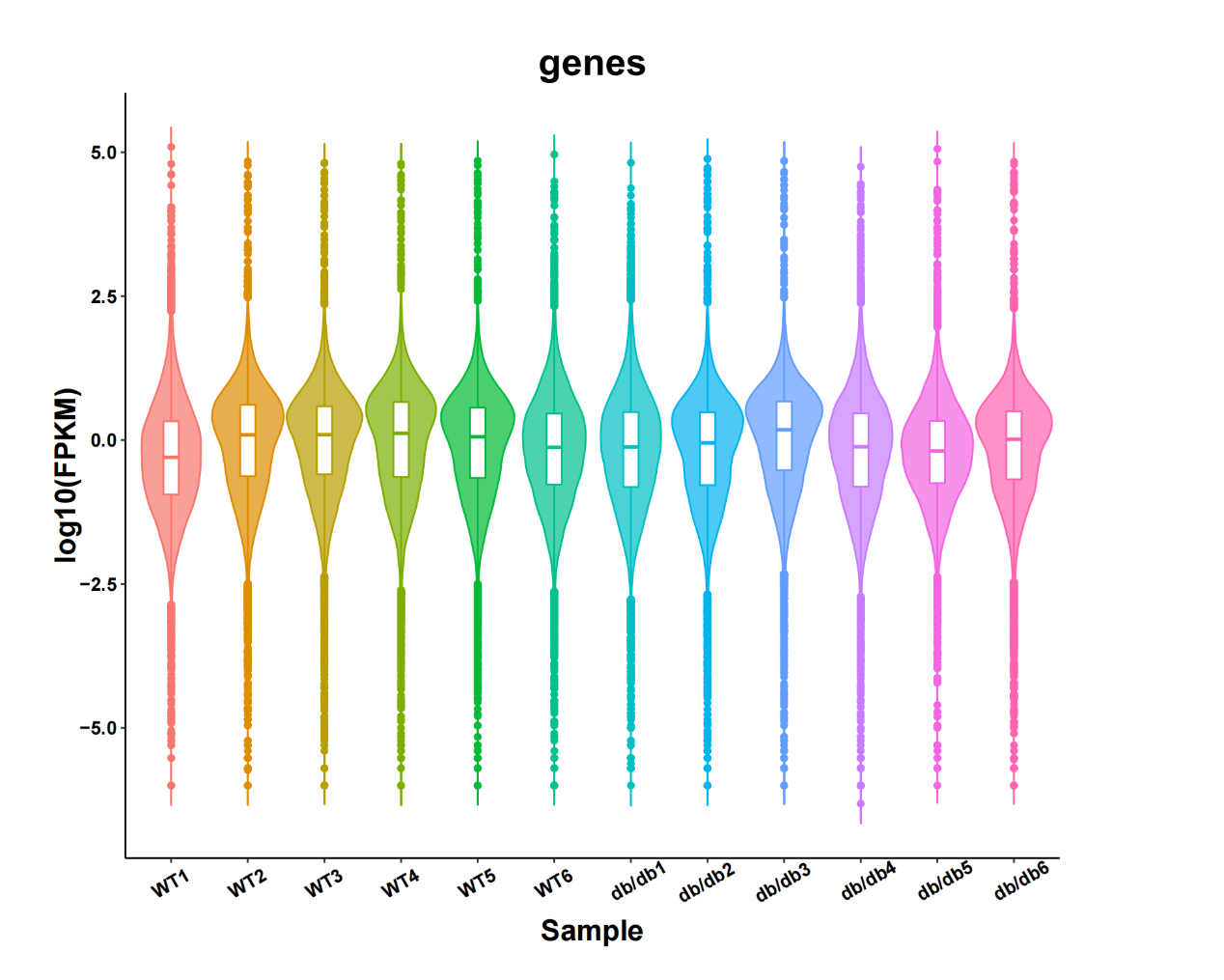


**Figure S1:** Statistical map of gene expression distribution in each sample


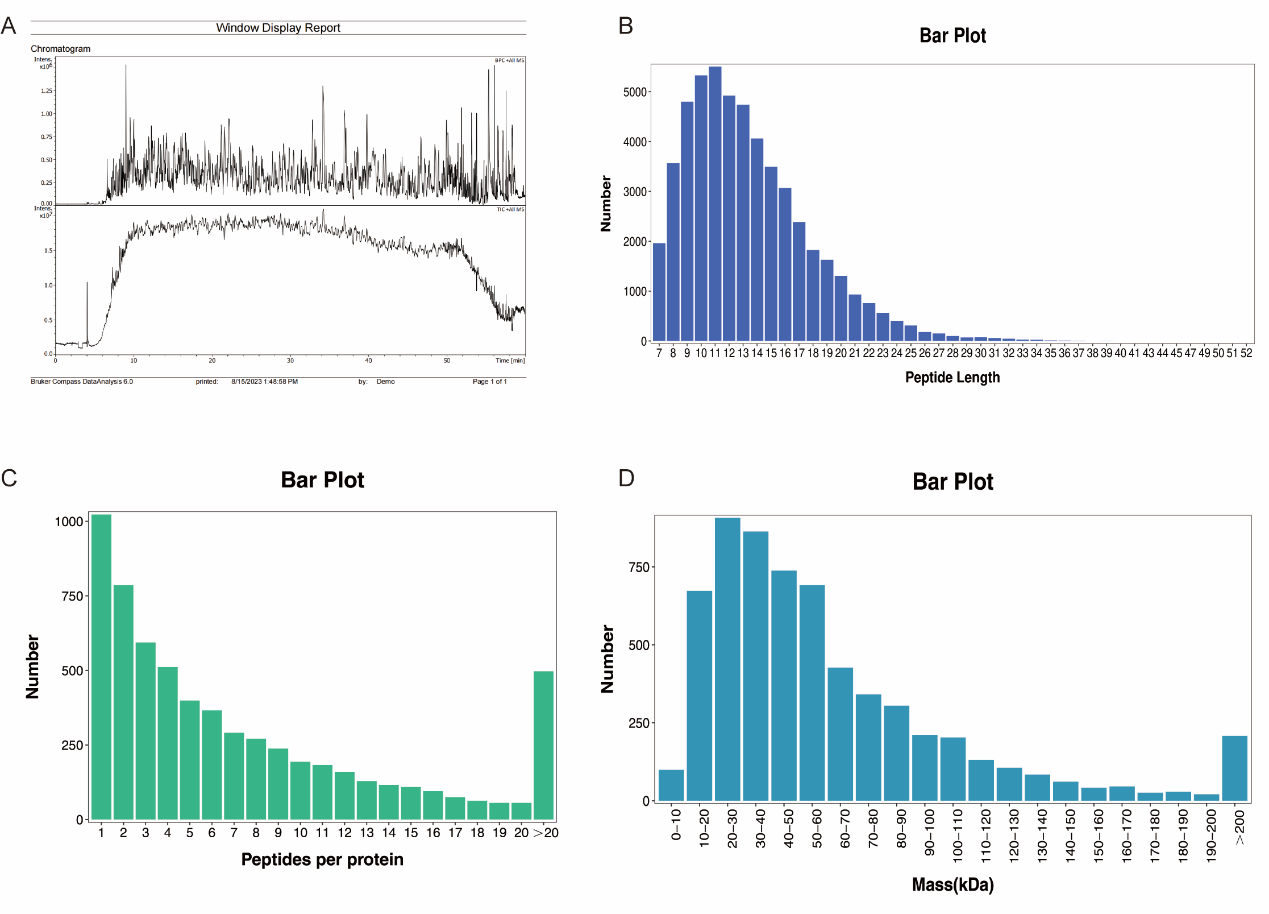


**Figure S2:** Reliability evaluation of proteomics of gastric proteomics in WT and db/db mice (A) TIC&BPC. (B) Length distribution of all identified peptides. (C) Distribution diagram of the number of peptides. (D) Distribution diagram of protein mass


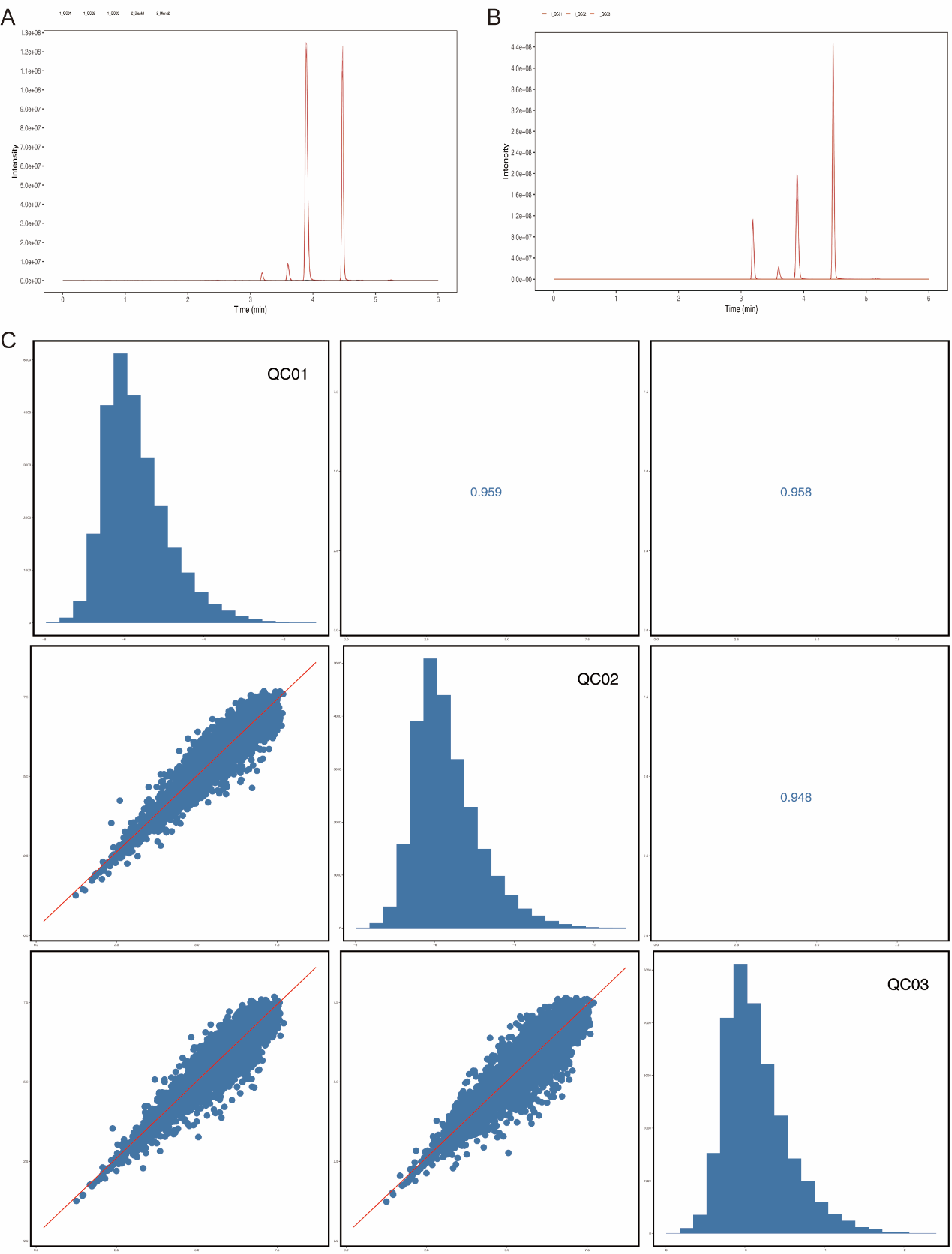


**Figure S3:**. Reliability evaluation of gastric metabonomics in WT and db/db mice. The overlaps of the spectral peak of three within-run QC samples in positive (A) and negative (B) mode. (C) Correlation map of QC samples


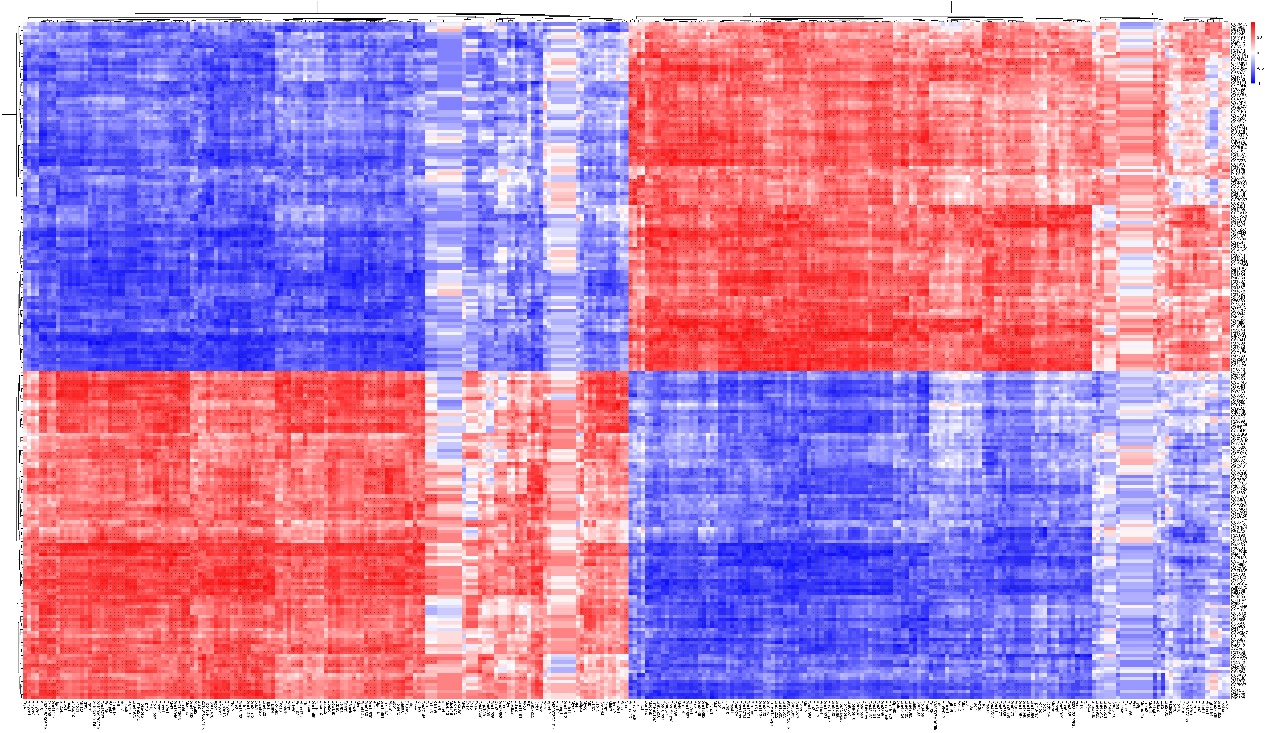


**Figure S4:** Spearman correlation analysis between all DEPs and DEGs


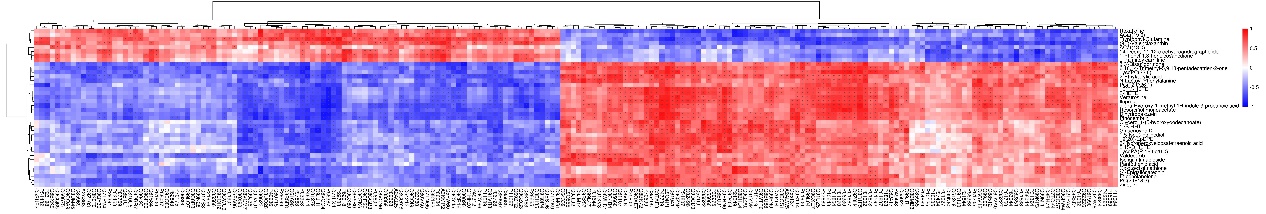


**Figure S5:** Spearman correlation analysis between all DEMs and DEPs
